# Syndromic obesity: clinical implications of a correct diagnosis

**DOI:** 10.1186/1824-7288-40-33

**Published:** 2014-04-02

**Authors:** Donatella Milani, Marta Cerutti, Lidia Pezzani, Pietro Maffei, Gabriella Milan, Susanna Esposito

**Affiliations:** 1 Pediatric Highly Intensive Care Unit, Department of Pathophysiology and Transplantation, Università degli Studi di Milano, Fondazione IRCCS Ca’ Granda Ospedale Maggiore Policlinico, Via Commenda 9, Milano 20122, Italy; 2 Department of Medicine, Padua University Hospital, Padua, Italy

**Keywords:** Alström syndrome, Bardet-Biedl syndrome, Cone-rod dystrophy, Obesity, Syndromic obesity

## Abstract

**Background:**

Although individual occurrence is rare, syndromic obesity with mental retardation has been reported in conjunction with 140 different diseases.

**Case presentation:**

The patient was born at term after a pregnancy complicated by threatened miscarriage. A diagnosis of Bardet-Biedl syndrome (BBS; OMIM #209900) was made in another hospital when she was 8 years old, but other clinical problems emerged subsequently. She came to our attention for the first time when she was 14 years old. The clinical picture, characterized by the presence of ophtalmological, renal, endocrinological, and liver disorders associated with a peculiar weight growth pattern, was more suggestive for Alström syndrome (ALMS; OMIM #203800); consequently, a genetic study was performed. Genetic analysis revealed a novel compound heterozygous frameshift mutation on exon 8 of ALMS1 (c. [3251_3258delCTGACCAG] and c. [6731delA]), which has not previously been described.

**Conclusion:**

Early onset of retinal degeneration associated with obesity represents a diagnostic challenge in paediatric and genetic practice, although the absence of skeletal abnormalities and developmental delay could help in addressing the clinical diagnosis. Confirmation of clinical suspicion by genetic analysis has been diriment in this case, since only a single gene is known to cause ALMS.

## Background

Obesity is a highly heritable complex disorder characterized by body mass index (BMI) >30 kg/m^2^ (Kg weight/m^2^ height) which poses a major threat to public health worldwide although it is seldom associated with metabolic syndromes [1]. Both isolated and syndromic obesities have been recognized; the latter are often associated with the presence of congenital malformations and neurological diseases (i.e., developmental delay and/or intellectual disability) [1,2]. Although individual occurrence is rare, syndromic obesity with mental retardation has been reported in conjunction with 140 different diseases. Some peculiar features can be found in syndromic obesity, such as retinal degeneration. This feature is of particular interest for its clinical relevance, rarity and diagnostic power; only 30 conditions have this association. Among these, Alström syndrome (ALMS; OMIM #203800) is an autosomal recessive multi-systemic disorder characterized by cone rod-dystrophy resulting in blindness in early childhood, progressive hearing impairment, infantile or adolescent onset dilated cardiomyopathy, metabolic defects leading to hyperinsulinemia, hypertriglyceridaemia, type-2-diabetes mellitus, and obesity [3], and progressive pulmonary, hepatic and renal dysfunction [4]. Diagnosis is very difficult in early infancy, due to age-dependent typical features, often not evident until 6–8 years (Table 1). The identification of ALMS as a ciliary protein disorder explains the range of observed phenotypes and their similarity to other ciliopathies [4] such as Bardet-Biedl syndrome (BBS; OMIM #209900), a multi-systemic disease that comprises retinal dystrophy associated to obesity, polydactyly, renal malformations, hypogonadism, and cognitive impairment [5]. BBS is an autosomal recessive condition, although an oligogenic mode of inheritance has been shown in some families where three mutations at two BBS loci are found [6,7]. Diagnosis may be considered at birth in children with polydactyly and renal abnormalities. However, because of the inconstancy of these two features, due to clinical variability, and the late onset of other symptoms (i.e. retinal degeneration, genital abnormalities, and obesity), diagnosis is usually given during later childhood.

**Table 1:** **Diagnostic criteria for Alström syndrome (modified from Marshall et al., 2007**[4]**) and for Bardet-Biedl syndrome (modified from Beales et al.,1999**[2]**)**

**ALMS**			**BBS**	
**Diagnosis is made when the following criteria are present:**			**Diagnosis is made when:**	
2 major OR 1 major + 2 minor			4 major criteria are present OR	
2 major OR 1 major + 3 minor			3 major plus 2 minor criteria are present	
2 major + 2 minor criteria OR 1 major + 4 minor criteria				
**Age**	**Major criteria**	**Minor criteria**	**Major criteria**	**Minor criteria**
<2 years	ALMS 1 mutation in 1 allele and/or family history of ALMS	Obesity	Rod-cone dystrophy	Speech disorder/delay
		DCM/CHF		Strabismus/cataracts/astigmatism
	Vision (nystagmus, photophobia)		Polydactyly	Brachydactyly/syndactyly
3-14 years	ALMS 1 mutation in 1 allele and/or family history of ALMS	Obesity and/or insulin resistance	Obesity	Developmental delay
			Learning	Polyuria/polidipsia (nephrogenic diabetes insipidus)
	Vision (nystagmus, photophobia, decreased acuity, cone dystrophy by ERG)	(History of) DCM/CHF	Disabilities	
		Hearing loss	Hypogonadism in males	Ataxia/poor coordination/imbalance
		Advanced bone age		
		Hepatic dysfunction	Renal anomalies	Mild spasticity (especially lower limbs)
		Renal failure		
				Diabetes mellitus
>15 years	ALMS 1 mutation in 1 allele and/or family history of ALMS	Obesity and/or insulin resistance and/or DM2		Dental crowding/hypodontia/small roots/high arched palate
	Vision (legal blindness, history of nystagmus in infancy/childhood, cone and rode dystrophy by ERG)	(History of) DCM/CHF		Left ventricular hypertrophy/congenital heart disease
		Hearing loss		
		Hepatic dysfunction		Hepatic fibrosis
		Renal failure		
		Short stature		
		Males-hypogonadism		
		Females-irregular menses and/or hyperandrogenism		

DCM/CHF: dilated cardiomyopathy with congestive heart failure; ERG: electroretinogram; DM2: type 2 diabetes mellitus.

To date, 18 genes (BBS1-18) are known to be associated with BBS [8], whereas only one gene has been identified for ALMS [5,9]. Nevertheless, overlapping phenotypes have been reported between ALMS and BBS [10-12].

In syndromes with overlapping clinical signs but different natural history, major complications and follow-up, differential diagnosis can be challenging in order to assess a specific management and treatment regimen. In this paper, we report a patient previously diagnosed with BBS who was reassessed for a clinical and genetic diagnosis of ALMS.

## Case presentation

Our patient is the only daughter of unrelated healthy parents, and family history was unremarkable for genetic diseases. She was born at term after a pregnancy complicated by threatened miscarriage. Her birth weight was 3,480 g, with a birth length of 48.5 cm, occipital frontal circumference of 33.5 cm, and APGAR score 9/10. Since the first months of life, nystagmus and exotropia were noticed, and a retinopathy with severe low vision (1/20) was subsequently diagnosed. Retinal hypopigmentation and peripheral atrophy with extension to macular region were detected in the first years of life, and lack of response was demonstrated with electroretinogram and visual evoked potentials. Leber’s congenital amaurosis was initially suspected. In addition, the pattern of growth was always over 2 standard deviations for weight centile. In accordance with the presence of these features, a diagnosis of BBS was made in another hospital when she was 8 years old. However, other clinical problems emerged subsequently. The ocular anterior segment was initially normal, but a left cortical cataract emerged when the patient was 12 years old. Moreover, biochemical investigation showed hypertriglyceridemia, high LDL-cholesterol, low HDL-cholesterol, and increased insulin and C-peptide levels with a normal value of hemoglobin A1c. Hepatic dysfunction with hypertransaminasemia and mild cholestasis was further investigated by ultrasound evaluation that showed liver steatosis. Mild hypertension and thickening of cardiac mass were detected shortly after by cardiologic examination, and mild micro-albuminuria and proteinuria were discovered by urine analysis. The patient was also treated for precocious puberty; after the stop of therapy, hirsutism, hyperadrogenism, and later oligoamenorrhoea were detected. She also suffered from scoliosis and restrictive pulmonary dysfunction. Additionally, bilateral sensorineural hearing loss for high frequencies was detected since she was 13 years old. No mental retardation or any signs of motor or speech delay were ever reported. A neurological evaluation was only requested for muscle cramps during adolescence; on that occasion, a suspicion of a mitochondrial disease was formulated due to the clinical spectrum of signs, but no imbalance of mitochondrial chain was confirmed.

**Figure 1:**
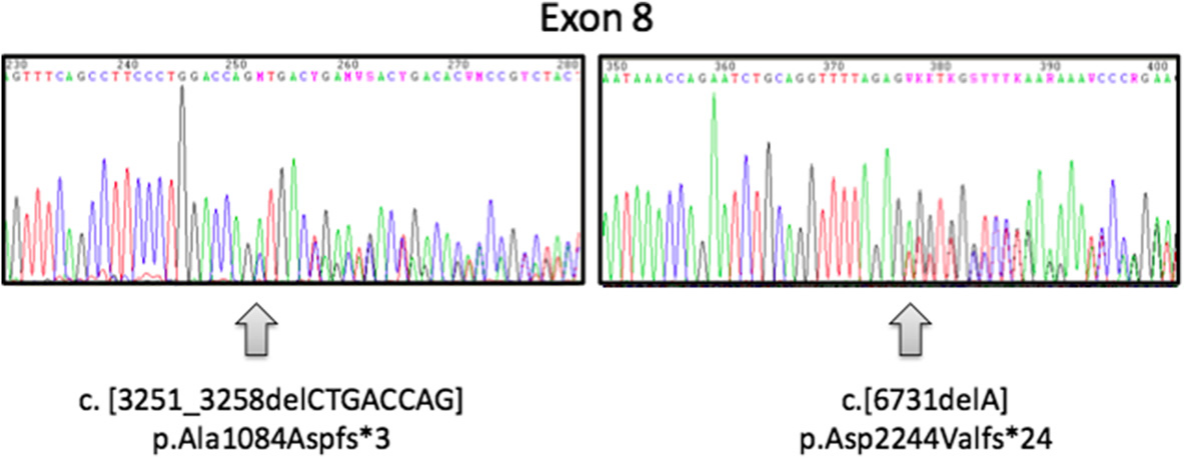
**Growth chart of the patient (four consecutive evaluations since the first visit in our center, BMI respectively 29.6, and after the beginning of an hypocaloric diet, 26.2, 27.1, 28.4 Kg/m**^**2**^**).**

**Figure 2:**
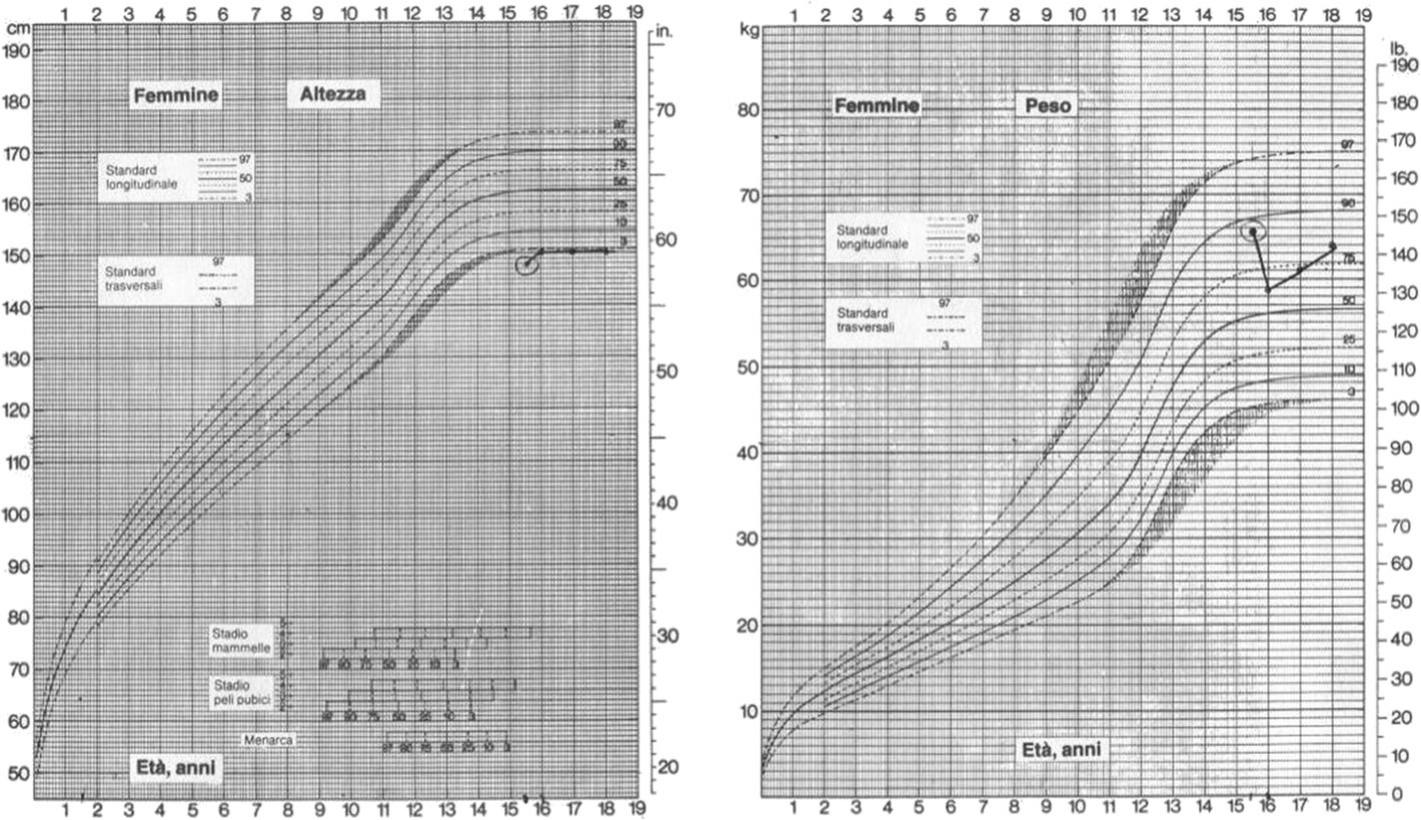
**Two novel heterozygous frameshift mutations were detected in the proband.** Panels show the chromatograms of the forward sequences relative to the regions on exon 8 in which the deletions (indicated by the arrows) are located, at positions 3251 and 6731 from the ATG of the coding sequence (c.) of *ALMS1* mRNA (NM_015120.4), respectively. The position of the frameshift (fs) and of the predicted premature termination codon (*) at the protein sequence level is indicated (p.). Nomenclature of mutations is according to den Dunnen JT and Antonarakis E [13].

She came to our attention for the first time when she was 14 years old. The clinical picture, characterized by the presence of ophthalmological, renal, endocrinological, and liver disorders associated with a peculiar weight growth pattern (Figure 1) was more suggestive of ALMS, and consequently a genetic study was performed. Genomic DNA, isolated from peripheral blood of the patient according to standard methods, was amplified using a standard polymerase chain reaction (PCR) protocol with HotStarTaq Master Mix Kit (QIAGEN GmbH, Hilden, Germany). Primer sequences and amplification conditions are available on request. PCR amplicons of exons 8, 10 and 16 of ALMS1 were purified, directly sequenced using ABI PRISM Big Dye Terminator Cycle sequencing Ready Reaction Kits, and analyzed with an ABI 3100 Sequencing Analyzer (Applied Biosystems, Forster City, CA, USA). Resulting sequences were compared with the GenBank mRNA reference sequence (NM_015120.4) using ClustalW2 (http://www.ebi.ac.uk/Tools/clustalw2/index.html). The nomenclature of mutations was assigned according to den Dunnen and Antonarakis [13]. Genetic analysis revealed a novel compound heterozygous frameshift mutation on exon 8 of *ALMS1* (c. [3251_3258delCTGACCAG] and c. [6731delA]; Figure 2), which has not been described previously. Both mutations result in a premature termination codon and truncation of the protein.

## Discussion

The range of biological causes that account for childhood obesity is heterogeneous, with several reports of multi-systemic monogenic disorders [14]. Many of these disorders have a peculiar presentation but could have an overlapping phenotype, indicating the likelihood of a shared common underlying pathway. This is well represented by BBS and ALMS, both classified as “ciliopathies” according to the aetiology. Clinical overlap between these two entities has been described, although BBS and ALMS are genetically distinct. In our patient there was no evidence of poly or syndactyly suggestive for BBS, and retinal dystrophy was observed since the first months of life. This stands in contrast with BBS, where retinal dystrophy is a nearly constant finding at over 5 years of age [6,10,15]. Moreover, complete blindness usually occurs in the second decade of life [16] in ALMS, and bilateral subcapsular cataracts are also common [17]. In our patient, sensorineural hearing loss for high frequencies, a peculiar feature described in 89% of individuals with ALMS [4], was also detected. The diagnosis of BBS was questioned by the presence of early onset retinal degeneration, insulin resistance, the absence of polydactyly, and relative preservation of cognitive function. Although ALMS may be difficult to diagnose because of its rarity and age dependent phenotype, early detection of the syndrome would allow clinicians to recognize the progressive development of a multi-organ pathology that can lead to reduced life expectancy [16], allowing for the establishment of a specific follow-up procedure and supportive therapy. In particular, the development of a heart failure, diabetes mellitus, obesity, liver and renal dysfunction could negatively interact, and they must be suspected and/or treated early.

## Conclusions

Early onset retinal degeneration associated with obesity represents a diagnostic challenge in paediatric and genetic practice, although the absence of skeletal abnormalities and developmental delay could facilitate clinical diagnosis. Careful medical history and clinical examination remain the cornerstones of diagnosis. Confirmation of clinical suspicion by genetic analysis has been diriment in this case since ALMS has been associated with a single causative gene, implying a faster diagnostic process with a lower economic impact. Within the last few years, research into the pathogenesis of ALMS has led to better management and treatment not only for this condition, but also for more common ciliopathies. Ultimately, the monogenic model of ALMS may facilitate the understanding of the biochemical pathways of more common diseases affecting the general population, such as obesity, diabetes, or single organ failure diseases.

## Consent

Written informed consent was obtained from the parents for publication of this Case report. A copy of the written consent is available for review by the Editor-in-Chief of this journal.

## Abbreviations

ALMS: Alström syndrome; BBS: Bardet-Biedl syndrome; BMI: Body mass index; DCM/CHF: Dilated cardiomyopathy with congestive heart failure; DM2: Type 2 diabetes mellitus; ERG: Electroretinogram; PCR: Polymerase chain reaction.

## Competing interests

The authors declare that they have no competing interests.

## Authors’ contributions

DM performed clinical diagnosis, and has been involved in drafting the manuscript, revising it critically for important intellectual content and providing final approval of the version to be published; MC has made substantial contributions to conception, acquisition of data, and has been involved in drafting the manuscript; LP has been involved in drafting the manuscript and revising it critically; PM and GM carried out the genetic analysis, clinical examination of the patient (PM), and a critical review of the manuscript; SE has been involved in revising the manuscript critically for important intellectual content and has given final approval of the version to be published. All authors have read and approved the final manuscript.
